# Integrating Morphological and Molecular Evidence Reveals a New Species and Two Synonyms in *Oreocharis* (Gesneriaceae)

**DOI:** 10.1002/ece3.73380

**Published:** 2026-04-06

**Authors:** Xi‐Zuo Shi, Zhi‐Xia Sun, Jia‐Xin Fu, Li‐Hua Yang

**Affiliations:** ^1^ Key Laboratory of National Forestry and Grassland Administration on Plant Conservation and Utilization in Southern China, South China Botanical Garden, Chinese Academy of Sciences Guangzhou China; ^2^ University of Chinese Academy of Sciences Beijing China; ^3^ College of Architecture and Civil Engineering, Sanming University Sanming China; ^4^ Fujian Provincial Key Laboratory of the Development and Utilization of Bamboo Resources, Sanming University Sanming China; ^5^ Guangxi Key Laboratory of Plant Conservation and Restoration Ecology in Karst & Terrain, Guangxi Institute of Botany, Guangxi Zhuang Autonomous Region and Chinese Academy of Science Guilin China; ^6^ National Gesneriaceae Germplasm Resources Bank of GXIB, Gesneriad Committee of China Wild Plant Conservation Association, Gesneriad Conservation Center of China (GCCC), Guilin Botanical Garden Chinese Academy of Sciences Guilin China; ^7^ State Key Laboratory of Plant Diversity and Specialty Crops, South China Botanical Garden, Chinese Academy of Sciences Guangzhou China

**Keywords:** morphological statistics, nuclear phylogeny, *Oreocharis*, taxonomic revisions, the broader Nanling mountains region

## Abstract

The genus *Oreocharis* is a species‐rich group within the family Gesneriaceae and represents an excellent model system for evolutionary studies. However, taxonomic understanding of this genus remains insufficient, resulting in several unresolved issues. In this study, we focus on two species groups within *Oreocharis*—the *O. argyreia* group and the *O. auricula* group—each of which presents distinct taxonomic challenges. To address these, we conducted detailed morphological comparisons and statistical analyses, along with phylogenetic analyses based on dense population‐level sampling. Our results support abolishing the variety *O. argyreia var. angustifolia* within *O. argyreia* but establishing a new species of *O. nanlingensis* to accommodate specimens of this variety collected from the broader Nanling mountains region. In addition, our findings suggest that *O. auricula var. denticulata* should be treated as a synonym of *O. magnidens* . We provide final taxonomic treatments, amended descriptions, photographs, and a distribution map for the relevant taxa.

## Graphical abstract

Using both population‐level phylogenetic analysis and morphological statistics, this study resolved several taxonomic problems within the genus *Oreocharis*. Our results support abolishing the variety *O. argyreia var. angustifolia*, establishing a new species *O. nanlingensis*, and treating *O. auricula var. denticulata* as a synonym of *O. magnidens*.

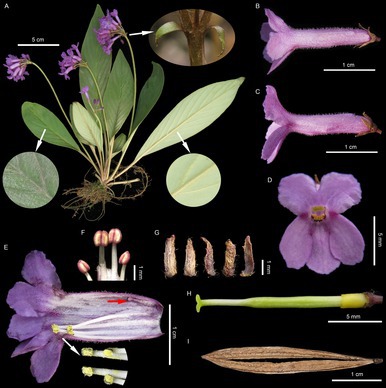

## 1 Introduction

Accurate species identification and delimitation is essential for ecology, conservation biology, and related basic sciences (Mace 2004). However, accomplishing this task effectively can be challenging under certain circumstances. Traditionally, plant identification and description have relied heavily on dried specimens. While this approach offers convenience, it can sometimes lead to misidentification due to morphological distortion in preserved material. Such errors are further compounded when only a limited number of specimens from a single population are examined. In addition, species identification and delimitation may be complicated by phenotypic plasticity and convergent evolution (Sultan 1987; Wake 2011). To minimize these inaccuracies, it is necessary to integrate morphological and molecular evidence obtained from extensive population‐level sampling (Hillis 1987; Schlick‐Steiner et al. 2010; Yang et al. 2019). The plant family Gesneriaceae is well known for its taxonomic complexity, particularly in the Old‐World subfamily Didymocarpoideae, where a number of misidentifications and erroneous delimitations have been documented in recent studies (e.g., Yang et al. 2019; Yang, Wen, et al. 2020; Yang, Feng, et al. 2020; Cai et al. 2020; Hong et al. 2020; Li et al. 2022; Olivar et al. 2022; Li et al. 2023; Liu, Gong, et al. 2025, Liu, Li, et al. 2025; Xiong et al. 2024, 2025). In this study, we focus on two species groups within the genus *Oreocharis*—the *O. argyreia* group and the *O. auricula* group—each of which presents distinct challenges in taxonomic treatment.

**FIGURE 1:**
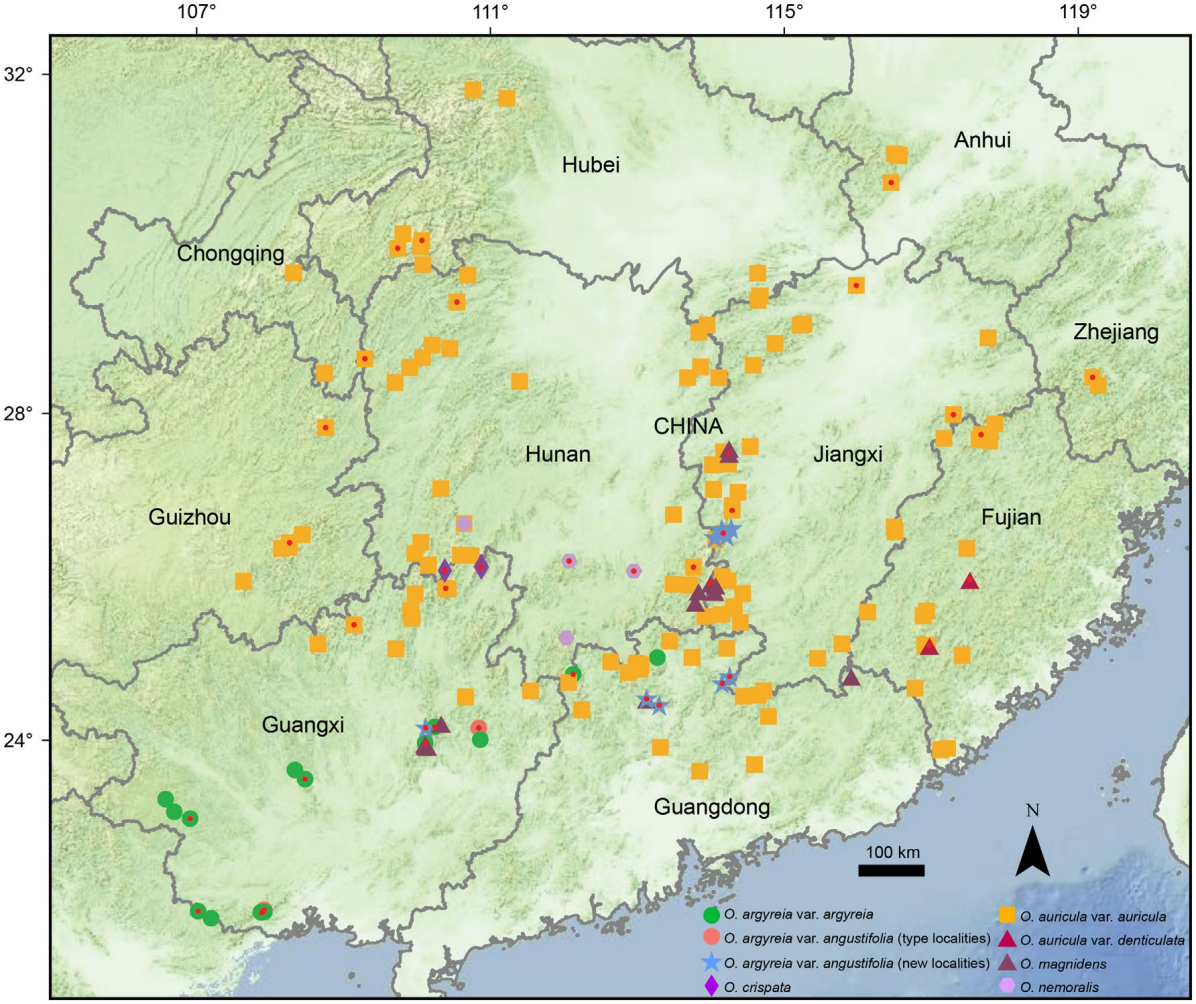
Geographical distribution of studied species. Red dots mark the sampling sites of the phylogenetic analysis.

*Oreocharis argyreia* Chun ex K.Y. Pan var. *argyreia* was first described by Pan in 1987 based on 11 collections from Guangxi and Guangdong, China, with the holotype originating from Jingxi Country, Guangxi (Figure S1; X.P. Gao 55553). In the same publication, Pan also described a new variety of this species, *O. argyreia var. angustifolia* K.Y. Pan, according to three collections from two localities in Guangxi. The holotype of this variety was collected from Shiwandashan mountains, Shangsi, Guangxi, China (Figure S2; W.T. Tsang 24218) and the paratypes were collected from Danaoshan mountains, Zhaoping, Guangxi, China (Figure S2; Y.K. Li 402493 and J.J. Wang 005338). Pan differentiated this new variety from the type variety mainly by the leaf blade length and width, i.e., *O. argyreia var. angustifolia* possess related narrow leaf blade compared to its original variety. In fact, the reliability of using continuous quantitative traits to diagnose new taxa depends on statistics of a large number of samples. However, a statistical analysis was lacking in Pan (1987). This new variety has been recollected only once (R.H. Liang LRH021, PE!) from its type locality since its publication, although it has been included in all subsequent literature and monographs (Wang et al. 1998; Li and Wang 2004). Nevertheless, this new variety was recently reported to be found at Jingangshan mountains, west of Jiangxi, China (Fan et al. 2014; Liao et al. 2024). Soon later, our field investigations found that the plants found in Jingangshan mountains also present in several other localities within the broader Nanling mountains region, spanning southwestern Jiangxi, southern Hunan, northern Guangdong, and eastern Guangxi (Figure 1). Our observations in field further found that the plants at these new distribution areas show obvious different flower characters from the plants at the type localities (Shiwangdashan moutains and Danaoshan moutains) of *O. argyreia var. angustifolia* . For example, plants from these new distribution areas possess obvious longer corolla lobes than plants from type localities. In particular, plants from these new distribution areas bloomed in April, while plants from type localities bloomed in September. This significant discontinuity of flowering phenology together with obvious morphological differences indicate that plants from these two regions might not represent the same species. This inference was further supported by our recent work on the molecular phylogeny of *Oreocharis*. Using 574 loci, Kong et al. (2022) reconstructed a robust phylogeny of *Oreocharis* including 111 species. Surprisingly, a sample (YLH494) collected from these new distribution areas (Shimentai Nature Reserve, Guangdong) did not cluster with the sample (YLH626) of *O. argyreia* that was collected from Damingshan mountains, Guangxi. Instead, this sample fell into a fully supported clade including *O. crispata* W.H. Chen & Y.M. Shui, *O. auricula* (S. Moore) C.B. Clarke, *O. nemoralis* Chun and *O. magidens* Chun ex K.Y. Pan, with a sister relationship to *O. crispata* . Collectively, these results indicate that the identification and delimitation within the *O. argyreia* species group might exist problems and need more detailed work to clarify them.

*Oreocharis auricula* (S. Moore) C.B. Clarke var. *auricula* was first described as a member of *Didymorcarpus* by Moore (1875), but had been subsequently transferred to *Oreocharis* by Clarke (1883). Möller et al. (2011) included another species, *O. sericea* H. Lév., into *O. auricula* possibly due to no substantially morphological differences between them. Currently, *O. auricula var. auricula* is the most widely distributed species in the genus, occurring throughout southern and southeastern China (Figure 1; Pan 1987; Wang et al. 1998; Li and Wang 2004). In 1987, Pan described a new variety in this species, *O. auricula var. denticulata* K.Y. Pan, based on a single specimen (Figure S3A; *Fujian Expedition Team 005367*) collected from Yong'an, Fujian, China. This new variety was included in all subsequent literature and monographs (Wang et al. 1998; Li and Wang 2004; Möller et al. 2011). This variety was represented by its type material, until two additional specimens were collected from Yong'an City during our field work, with one from its type locality and the other from a near site (Figure 1). Pan (1987) realized that this variety could be distinguished from the type variety, *O. auricula var. auricula* , by its dentate leaf blade margin (vs. inconspicuously crenate to subentire). In fact, the variety *O. auricula var. denticulata* can also be easily differentiated from the type variety by its bullate leaf blade (vs. smooth) and white villous on its adaxial leaf blade (vs. sericeous to glabrescent), which were overlooked by Pan (1987). In the same publication of *O. auricula var. denticulata* , Pan (1987) also described another new species, i.e., *O. magnidens* Chun ex K.Y. Pan. The author compared *O. magnidens* to *O. auricula var. auricula* , and found that *O. magnidens* differs from the latter in its bullate leaf blade (vs. smooth) and white villous on its adaxial leaf blade (vs. sericeous to glabrescent). However, Pan (1987) missed a comparison between *O. magnidens* and *O. auricula var. denticulata* , despite that the types of these two species are nearly the same (Figure S3). It is not clear why Pan (1987) did not compare *O. magnidens* with *O. auricula var. denticulata* . *Oreocharis magnidens* was once regarded as an endemic species restricted to the Dayaoshan mountains in Guangxi (Pan 1987; Wang et al. 1998; Li and Wang 2004), while *O. auricula var. denticulata* was endemic to Yong'an City, Fujian. This pronounced disjunction in their reported distributions likely have led Pan to treat them as distinct taxa at that time. However, recent field works revealed that *O. magnidens* is a widespread species with numerous records found at the broader Nanling mountains region, spanning southern Guangxi and Hunan, northern Guangdong and southwestern Jiangxi (Figure 1; Zheng and Xia 2002; Guo et al. 2017; Feng et al. 2018). These new discoveries make the geographic distributions of *O. magnidens* and *O. auricula var. denticulata* become continuous, and also lead to the necessity of reconsideration of the taxonomic treatments among these taxa.

In recent years, we have conducted extensive field investigations on Gesneriaceae in south China. These efforts have yielded a wealth of data and numerous samples to address the issues noted above. In this study, we therefore aim to resolve the taxonomic problems within the *O. auricula* and *O. argyreia* species groups by integrating morphological comparisons with molecular phylogenetic analyses based on population‐level sampling. Our comprehensive approach confirms the recognition of one new species and two new synonyms, which are formally presented below.

## 2 Materials and Methods

### 2.1 Morphological Observation and Analysis

To perform detailed morphological observation and comparison for these studied and related species, we carried out extensive field investigations from 2018 to 2025. During these field surveys, we observed each taxonomic character from living plants in every investigated population, and took photographs for each observed individual. We also checked specimens of the related species housed in IBSC, PE, KUN, IBK, SYS and GXMI, as well as specimen photos deposited in several web databases, such as Chinese Virtual Herbarium (http://www.cvh.ac.cn), Kew Herbarium (http://apps.kew.org/herbcat/navigator.do), Royal Botanical Garden Edinburgh Herbarium (https://data.rbge.org.uk/search/herbarium) and Muséum d'Histoire naturelle (https://science.mnhn.fr/institution/mnhn/search). In addition, we carried out a comprehensive literature study, including all relevant monographs (Wang et al. 1998; Li and Wang 2004; Liao et al. 2024) and recently published literature (e.g., Pan 1987; Zheng and Xia 2002; Guo et al. 2017; Feng et al. 2018; Fan et al. 2014; Xiong et al. 2024, 2025; Li et al. 2025; Nguyen et al. 2025; Tang et al. 2025; Xie et al. 2025).

Given that the key differences between *O. argyreia var. argyreia* and *O. argyreia var. angustifolia* are quantitative traits (i.e., leaf blade length, leaf blade width, and their ratio), we further performed a statistical analysis for these taxa. The analysis was based solely on live plants collected in the field, as quantitative traits can change significantly after prolonged cultivation or dehydration. We firstly photographed all collected leaves alongside a scale bar at field. From each population, three to fifteen individuals were selected, and one mature leaf per individual was photographed for subsequent measurement. Using the software ImageJ (Abràmoff et al. 2004), we then measured the leaf blade length (LbL) and width (LbW) from these images and calculated the leaf blade length‐to‐width ratio (rLW) for each sample (Table S1). Thereafter, we conducted a one‐way analysis of variance (ANOVA) for these three traits in R v4.3.1 (R Core Team 2022), followed by Tukey's HSD post hoc test for pairwise comparisons among taxa. In addition, we performed a principal component analysis (PCA) using the *prcomp* function in R on the same three traits used in the ANOVA. The resulting first two principal components were visualized as a scatter plot using the package ggplot2 v3.5.1 (Wickham 2016).

### 2.2 Taxon Sampling, DNA Exaction, Sequencing, and Phylogenetic Analysis

To perform the phylogenetic analysis, we sampled all studied and another phylogenetically related species according to the phylogeny of *Oreocharis* estimated in Kong et al. (2022). For each of these species, we sampled multiple populations to represent its whole geographic distribution (Figure 1). These included 15 populations of *O. auricula var. auricula* , 9 populations of *O. argyreia var. angustifolia* , 7 populations of *O. argyreia var. argyreia* , 5 populations of *O. magnidens* , 3 populations of *O. crispata* , 2 populations of *O. auricula var. denticulata* , and 2 populations of *O. nemoralis* . In addition, we sampled two species of *Palmatiboea* (Liu, Li, et al. 2025) as outgroups according to the phylogeny of Gesneriaceae estimated in Yang et al. (2023). In each population, we collected leaf materials for 1–7 individuals (Table S2). Therefore, a total of 59 individuals from 46 populations were collected for the phylogenetic analysis in this study.

We sequenced seven single copy nuclear genes (47, 72, 97, 100, 111, 115 and 165) and the internal transcribed spacer (ITS) region. The seven single copy loci were initially developed from 11 *Primulina* transcriptomes (Ai et al. 2015), and had been demonstrated to be useful for resolving phylogenetic relationships within *Oreocharis* (Sun et al. 2019, 2024). The methods of DNA extraction, PCR amplification, sequence assembly and sequence alignment followed our previous studies (Yang, Wen, et al. 2020; Sun et al. 2024; Shi et al. 2024). All newly generated sequences had been deposited in GenBank with their accession numbers can be found in Table S2.

We inferred phylogenies using both Maximum Likelihood (ML) and Bayesian Inference (BI) methods based on a combined matrix of all these eight loci. For the ML analysis, we choose the IQ‐TREE v2.1.4 (Nguyen et al. 2015). In IQ‐TREE, we identified the optimal partitioning scheme and selected the best‐fitting substitution model for each partition using ModelFinder (Kalyaanamoorthy et al. 2017). Node support for the ML phylogeny was assessed with 1000 ultrafast bootstrap (UFBoot) replicates. For the BI analysis, we used MrBayes 3.2.7 (Ronquist et al. 2012) under the same partitioning scheme as in the ML analysis. Because MrBayes does not implement all the complex models evaluated in ModelFinder, we applied the nearest available model in MrBayes for each partition. In MrBayes, we ran two independent Markov chain Monte Carlo (MCMC) analyses, each consisting of one cold chain and three heated chains, for 10,000,000 generations. Convergence was assessed by ensuring the potential scale reduction factor approached 1 and the standard deviation of split frequencies fell below 0.01. A majority‐rule consensus tree was generated after discarding the first 25% sampled trees as burn‐in. All resulting trees were visualized in FigTree v1.4.4 (A. Rambaut, University of Edinburgh, http://tree.bio.ed.ac.uk/software/figtre).

## 3 Results and Discussion

### 3.1 Morphological Affinities

**FIGURE 2:**
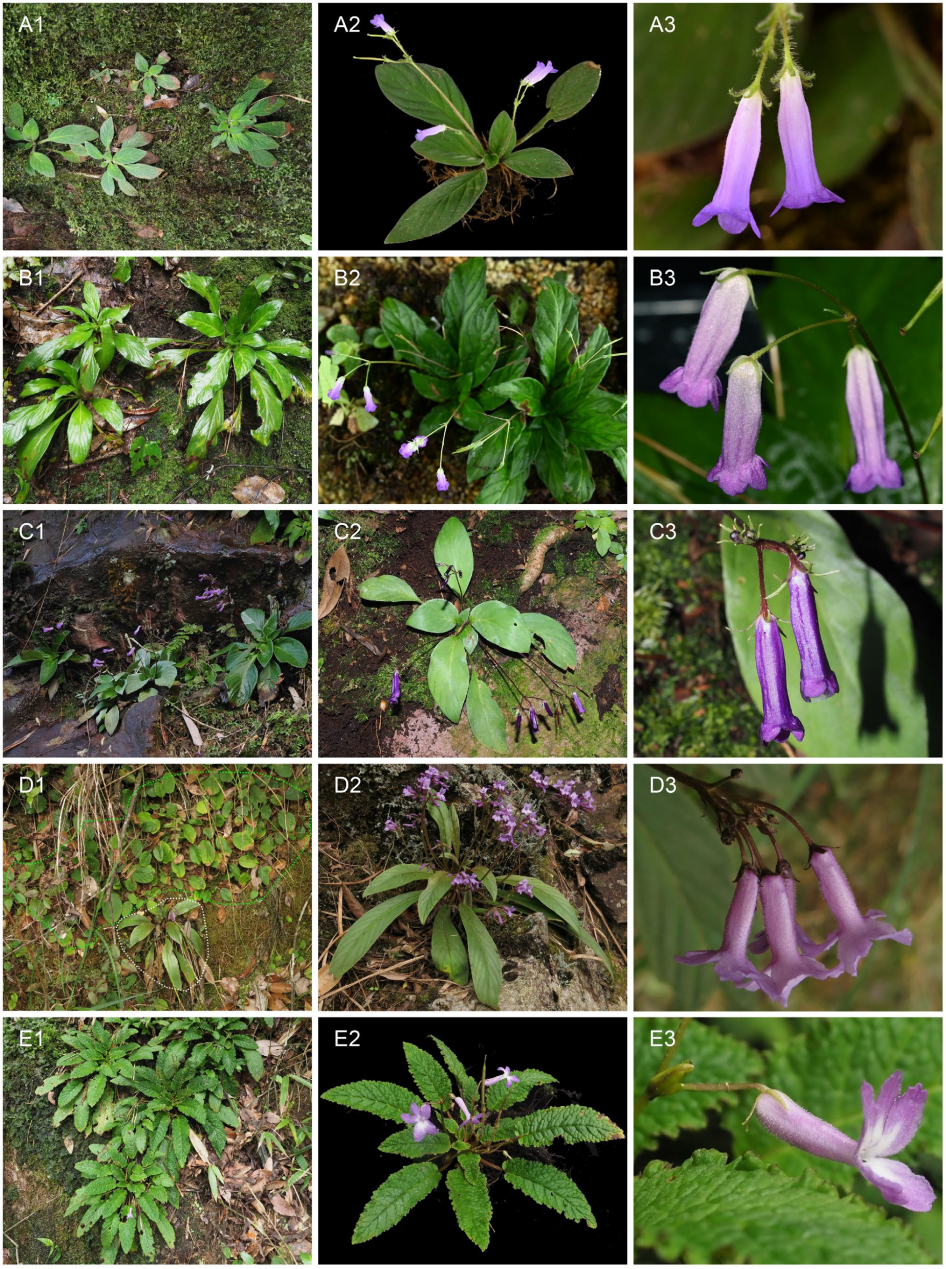
Morphological comparisons among *Oreocharis argyreia var. argyreia* , *O. argyreia var. angustifolia* and *O. crispate*. (A) Photographs of *O. argyreia var. angustifolia* were taken from its holotype locality (Shiwandashan mountains, Guangxi). (B) Photographs of *O. argyreia var. angustifolia* were taken from its paratype locality (Danaoshan mountains, Shangsi, Guangxi). (C) Photographs of *O. argyreia var. argyreia* were taken from Damingshan moutains, Wuming, Guangxi. (D) Photographs of *O. argyreia var. angustifolia* were taken from Ciping, Jinggangshan, Jiangxi. In D1, the green dashed box highlights *Oreocharis auricula* , while the red dashed box outlines *O. argyreia var. angustifolia* . (E) Photographs of *O. crispata* were taken from Caiwan, Quanzhou, Guangxi.

**FIGURE 3:**
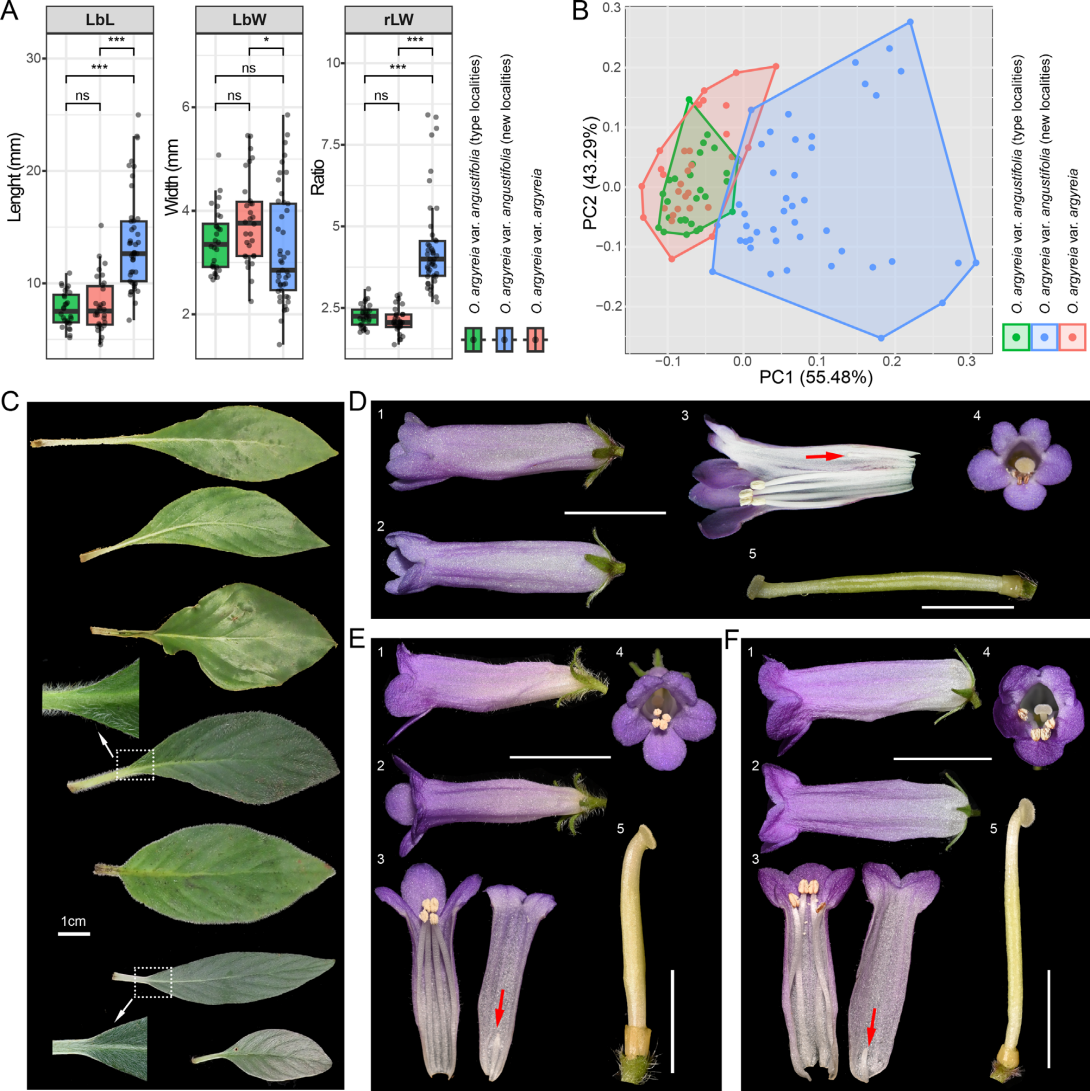
Morphological comparisons between *Oreocharis argyreia var. angustifolia* and *O. argyreia var. argyreia* . (A) One‐way ANOVA statistics of three leaf blade characters (i.e., LbL, LbW and rLW). Statistical significance from the Tukey HSD post hoc test is indicated as follows: Ns, not significant; *, *p* < 0.05; ***, *p* < 0.001. (B) The scatter plot based on the first two PCA axes of three leaf blade characters. (C) Leaf blade variation of *O. argyreia var. angustifolia* within its type localities (Shiwandashan and Danaoshan). (D) Flower photographs of *O. argyreia var. argyreia* from Damingshan mountains, Wuming, Guangxi. (E) Flower photographs of *O. argyreia var. angustifolia* from its holotype locality (Shiwandashan mountains, Guangxi). (F) Flower photographs of *O. argyreia var. angustifolia* from its paratypes locality (Danaoshan mountains, Shangsi, Guangxi). (1) flowers in side view; (2) flowers in top view; (3) opened flower showing stamens, staminodes (red arrows) and corolla inside; (4) flowers in top view; (5) pistil. Scale bars: 1 cm for flowers, 0.5 cm for pistil.

Our detailed morphological comparisons based on both herbarium specimens and living materials suggested that the plants from the broader Nanling mountains region might had been misidentified as *O. argyreia var. angustifolia* in previous studies (e.g., Fan et al. 2014; Kong et al. 2022; Xu et al. 2023; Liao et al. 2024). These plants displayed a salverform corolla with obviously long lobes, bilobed stigma and much longer leaf blade with 9–15 pairs of lateral veins (Figure 2D), while plants from the type localities (i.e., Southern Guangxi) of both *O. argyreia var. angustifolia* and *O. argyreia var. argyreia* show a tubular corolla with short lobes, disc‐shaped stigma and much shorter leaf blade with 5–7 pairs of lateral veins (Figure 2A–C). In particular, the scatter plot based on the first two PCA axes of three leaf blade characters (i.e., LbL, LbW and rLW) showed obvious discontinuity between plants from the broader Nanling mountains region and other areas of *O. argyreia* (Figure 3B). Statistical analyses based on 103 samples (30 individuals of *O. argyreia var. argyreia* , 28 individuals of *O. argyreia var. angustifolia* from the type localities and 45 individuals of *O. argyreia var. angustifolia* from the new areas; Table S1) also support our observations. All three measured characters show statistically significant differences between plants from the new areas and the type areas (Figure 3A; Table S3).

In fact, the salverform corolla with obviously longer lobes of the plants of *O. argyreia var. angustifolia* from the broader Nanling mountains region suggests an affinity with species of the *O. auricula* group, especially *O. crispata*. Nevertheless, these plants can be easily distinguished from *O. crispata* by their smooth leaf blade with an entire margin (vs. rugose leaf blade with crispate and irregularly dentate margin), as well as the bilobed stigma (vs. disc‐shaped). The gross morphology of the plants of *O. argyreia var. angustifolia* from the broader Nanling mountains is also similar to *O. auricula var. auricula* , but can be distinguished from the latter by its much narrower leaf blade with 9–15 pairs of lateral veins and the bilobed stigma (vs. disc‐shaped). Moreover, in our field investigations, we found the plants of *O. argyreia var. angustifolia* from the broader Nanling mountains can grow together with the plants of *O. auricula var. auricula* (Figure 2D1), but keep their morphological integrity respectively. This finding suggested that the two groups of plants have established complete reproductive isolation between them and thus represent two well biological species (Mayr 1992).

In contrast, we found that the plants of *O. argyreia var. angustifolia* from its type localities are indistinguishable from the plants of *O. argyreia var. argyreia* . Pan (1987) considered that *O. argyreia var. angustifolia* can be mainly distinguished from the type variety by its narrow leaf blade. However, during our visit to the type localities of *O. argyreia var. angustifolia* , we found that the leaf blade shape of this variety exhibits extensive variation, with many individuals being indistinguishable from the type variety *O. argyreia var. argyreia* (Figure 3C). To confirm whether there is continuous variation in leaf blade characters between *O. argyreia var. argyreia* and *O. argyreia var. angustifolia* , we carried out a statistical analysis comparing these two varieties using three characters. The results showed that the ranges of all three characters overlapped extensively between samples of *O. argyreia var. argyreia* and samples of *O. argyreia var. angustifolia* from its type locations. For example, the LbL, LbW, and rLW of *O. argyreia var. angustifolia* from its type locations range from 5.2 to 10.9, 2.7 to 5.1 and 1.7 to 3.1, respectively, and range from 4.5 to 15.1, 2.3 to 5.5 and 1.4 to 3.0, respectively, within *O. argyreia var. argyreia* . Tukey's HSD post hoc test further confirmed that there were no significant differences between *O. argyreia var. argyreia* and *O. argyreia var. angustifolia* from the type localities for any of the three characters (LbL: *p* = 0.899; LbW: *p* = 0.242; rLW: *p* = 0.849; Figure 3A and Table S3). Furthermore, within the scatter plot based on the first two PCA axes of these three characters, samples of *O. argyreia var. angustifolia* from its type localities fully fell into the space of *O. argyreia var. argyreia* . These results together suggest that all the LbL, LbW and rLW cannot be served as reliable diagnostic characters between *O. argyreia var. angustifolia* and *O. argyreia var. argyreia* . In addition, Pan (1987) recognized that *O. argyreia var. angustifolia* can differ from the type variety by its pubescent ovary. However, our examination of the living plants from its type locality revealed that the ovaries of *O. argyreia var. angustifolia* are glabrous (Figure 3D–F), suggesting that the earlier description based on dry specimens was likely inaccurate.

**FIGURE 4:**
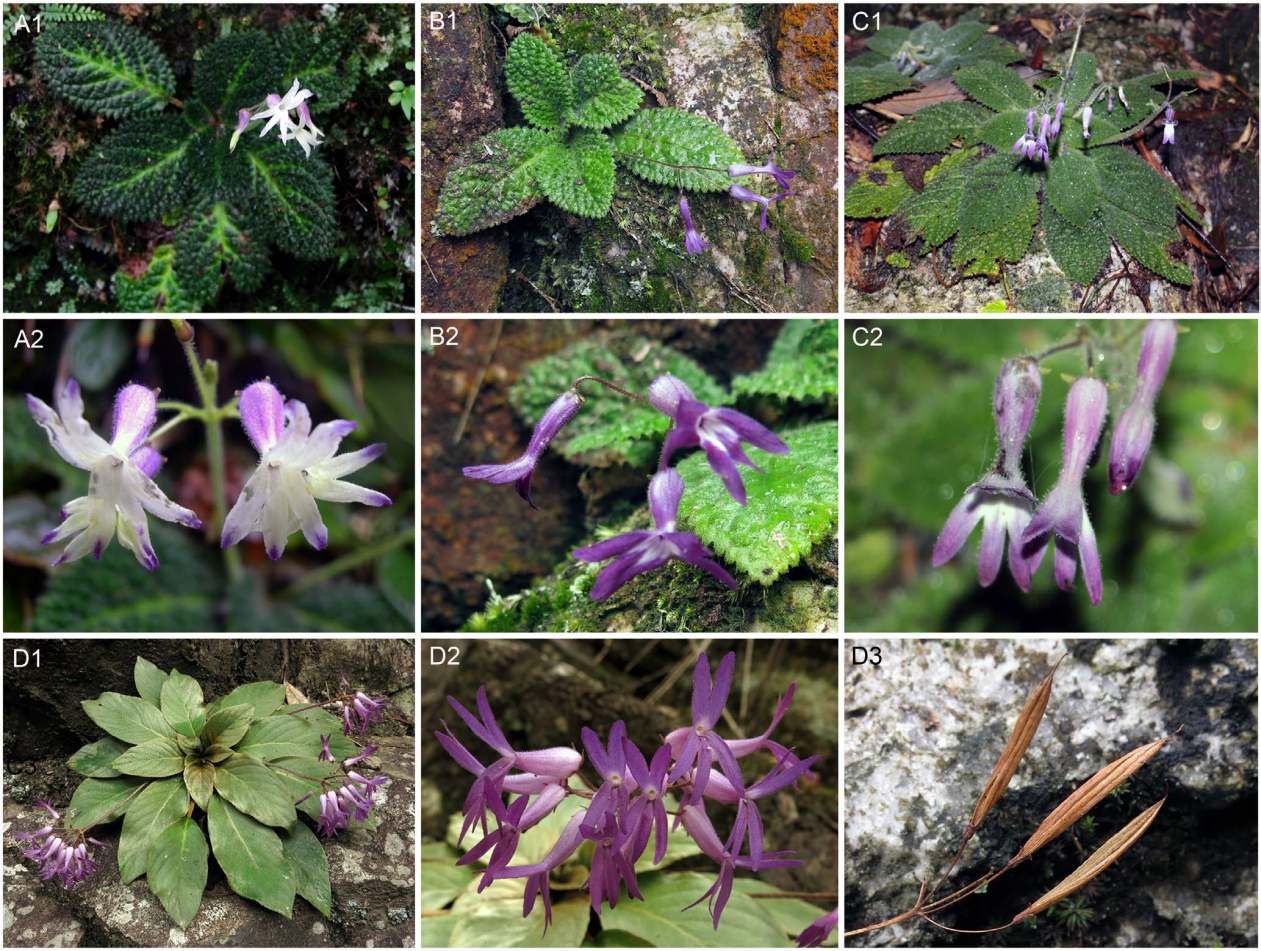
Morphological comparisons among *Oreocharis magnidens* , *O. auricula var. denticulata* and *O. auricula var. auricula* . (A) Photographs of *O. magnidens* were taken from Shengtangshan Mountains, Guangxi. (B) Photographs of *auricula* var. *denticulata* were taken from Jiulong Village, Yong'an, Fujian. (C) Photographs of *O. magnidens* were taken at Ruyuan Canyon, Guangdong. (D) Photographs of *O. auricula var. auricula* were taken from Wuyishan Nature Reserve, Fujian. (1) habitat; (2) flower; (3) fruit.

Our detailed morphological observation also showed that there were no essential differences between *O. magnidens* and *O. auricula var. denticulata* (Figure 4). However, both *O. magnidens* and *O. auricula var. denticulata* can be easily differentiated from *O. auricula* based on their bullate leaf blade, obviously dentate leaf blade margin, and white villous on the adaxial leaf blade (vs. smooth leaf blade, crenate to subentire leaf blade margin, and sericeous to glabrescent on adaxial leaf blade).

The detailed morphological comparisons among all these species are provided in Table 1.

**TABLE 1:** Morphological comparisons among *Oreocharis nanlingensis*, *O. crispata* , *O. argyreia* , *O. auricula* and *O. magnidens* .

Characters	*O. nanlingensis*	*O. crispata*	*O. argyreia*	*O. auricula*	*O. magnidens*	*O. auricula var. denticulata*
Leaf blade	Smooth with entire margin; adaxially densely appressed pubescent	Rugose with crispate and irregularly dentate margin; adaxially pubescent	Smooth with entire margin; adaxially densely white villous or pubescent	Smooth with serrate to subentire margin; adaxially sericeous to glabrescent	Bullate with dentate to crenate margin; adaxially white villous	Bullate with dentata to crenate margin; adaxial white villous
Number of veins	9–15 pairs	8–9 pairs	5–7 pairs	5–8 pairs	5–9 pairs	5–8 pairs
Corolla	Salverform	Salverform	Tubular	Salverform	Salverform	Salverform
Corolla limb	Conspicuous 2‐lipped, lobes oblong to broadly oblong, apex rounded	Slightly 2‐lipped, lobes oblong, apex rounded	Slightly 2‐lipped, lobes semi‐orbicular, apex rounded	Conspicuous 2‐lipped, lobes narrowly oblong, apex acute	Conspicuous 2‐lipped, lobes narrowly oblong, apex acute	Conspicuous 2‐lipped, lobes narrowly oblong, apex acute
Corolla tube	1.6–1.7 cm long, slightly constricted at throat	ca. 1.3 cm long, slightly constricted at throat	1.4–2 cm long, gradually ampliative from base to mouth	1.2–1.5 cm long, strongly constricted at throat	ca. 1.2 cm long, strongly constricted at throat	ca. 1.2 cm long, strongly constricted at throat
Pistil	1.5–1.6 cm	ca. 1.1 cm	0.9–1.8 cm	0.8–1.3 cm	ca. 0.8 cm	ca. 0.8 cm
Stigma	Bilobed	Disc‐shaped	Disc‐shaped	Disc‐shaped	Disc‐shaped	Disc‐shaped
Flowering time	March to April	April to May	August to October	May to September	June to August	June to August

### 3.2 Phylogenetic Relationships

**FIGURE 5:**
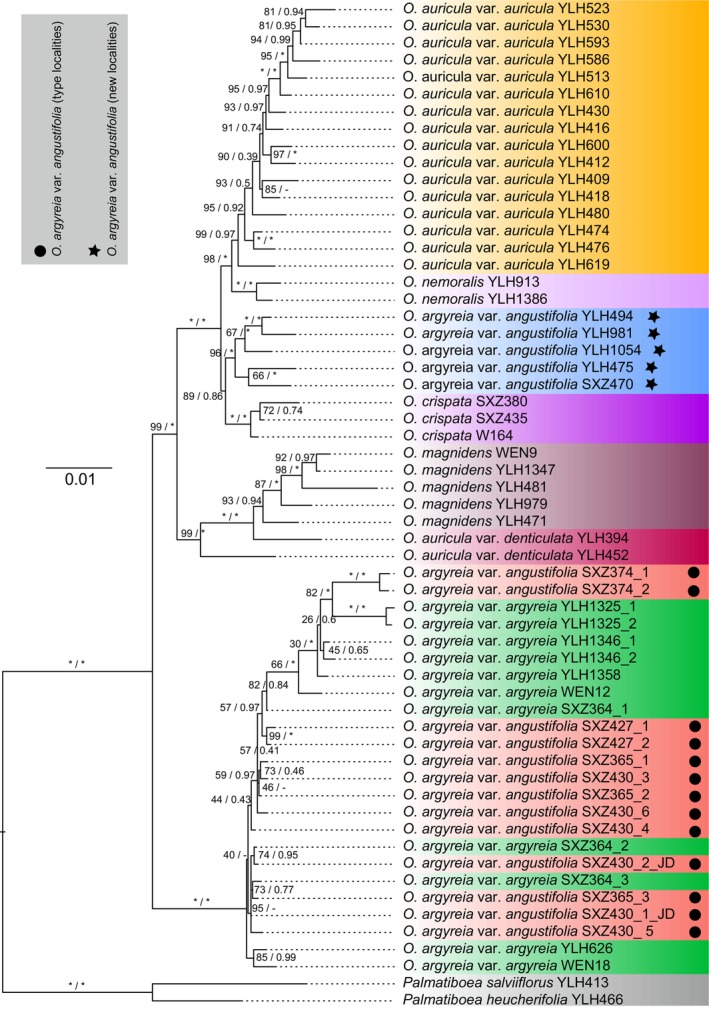
Maximum likelihood (ML) tree inferred from the combined eight nuclear loci dataset. The support values based on 1000 ultrafast bootstrap (UFBoot) replicates are shown on each branch. For comparison, Bayesian inference (BI) posterior probabilities (PP) are provided alongside the UFBoot values. Asterisks denote maximum support (UFBoot = 100, PP = 1.0), while dashes indicate conflicts between the ML and BI topologies. Star after the species name indicate that the sample was collected from the new localities of *O. argyreia var. angustifolia* .

To confirm our inferences based on morphological comparisons, we further performed phylogenetic analyses based on seven single‐copy nuclear loci and the ITS region for 59 individuals from 46 populations. The combined alignment of these eight loci comprises a total of 4313 nucleotide bases, of which 483 (11.1%) are parsimony‐informative sites. The characteristics of each locus are provided in Table 2. Both ML and BI analyses of the combined matrix showed largely congruent topologies, with only few conflicts at shallow nodes (Figure 5). However, quite a few nodes possess low supports (UFBoot < 50 or PP < 0.5) in both ML and BI phylogenies. Despite this, we found that all samples of *O. argyreia var. argyreia* , together with the samples of *O. argyreia var. angustifolia* from its type localities (i.e., southern Guangxi), form a fully supported clade (UFBoot = 100, PP = 1; Figure 5). Nevertheless, monophyly of neither of the two varieties was recovered in the phylogenies. In addition, all samples of *O. argyreia var. angustifolia* from the new areas (i.e., the broader Nanling mountains region) form a highly supported monophyly in both ML and BI phylogenies (UFBoot = 96, PP = 1; Figure 5), and, as expected, this clade fell into the *O. auricula* species group clade rather the clade containing the samples of *O. argyreia var. angustifolia* from its type localities. Within the *O. auricula* species group clade, all current recognized species formed a fully or highly supported monophyly, except for *O. auricula* . Samples of the type variety *O. auricula var. auricula* form a highly supported clade (UFBoot = 99, PP = 1; Figure 5), while the two samples of the other variety *O. auricula var. denticulata* did not cluster with them. In contrast, the two samples of *O. auricula var. denticulata* together with all samples of *O. magnidens* form a highly supported monophyly (UFBoot = 99, PP = 1; Figure 5).

**TABLE 2:** Summary of the alignments used in the phylogenetic analyses.

Loci	Number of accessions	Alignment length (bp)	Constant sites (bp)	Parsimony‐informative sites (bp)
47	59	194	136	32
72	59	427	358	42
97	44	697	587	65
100	58	502	375	73
111	59	775	687	46
115	58	340	269	46
165	59	647	559	50
ITS	44	731	517	129
Combined	59	4313	3488	483

Therefore, integrating both morphological and phylogenetic evidence, our study supports the removal of the variety *O. argyreia var. angustifolia* from *O. argyreia* . However, specimens previously identified as *O. argyreia var. angustifolia* from the broader Nanling mountains region should be recognized as a distinct new species. In addition, our results support treating *O. auricula var. denticulata* as a synonym of *O. magnidens* . These taxonomic revisions are formally presented below.

## 4 Taxonomic Treatments

### 4.1 *Oreocharis nanlingensis* X.Z. Shi & Li H. Yang, sp. nov. (Figures 6 and S4)

**FIGURE 6:**
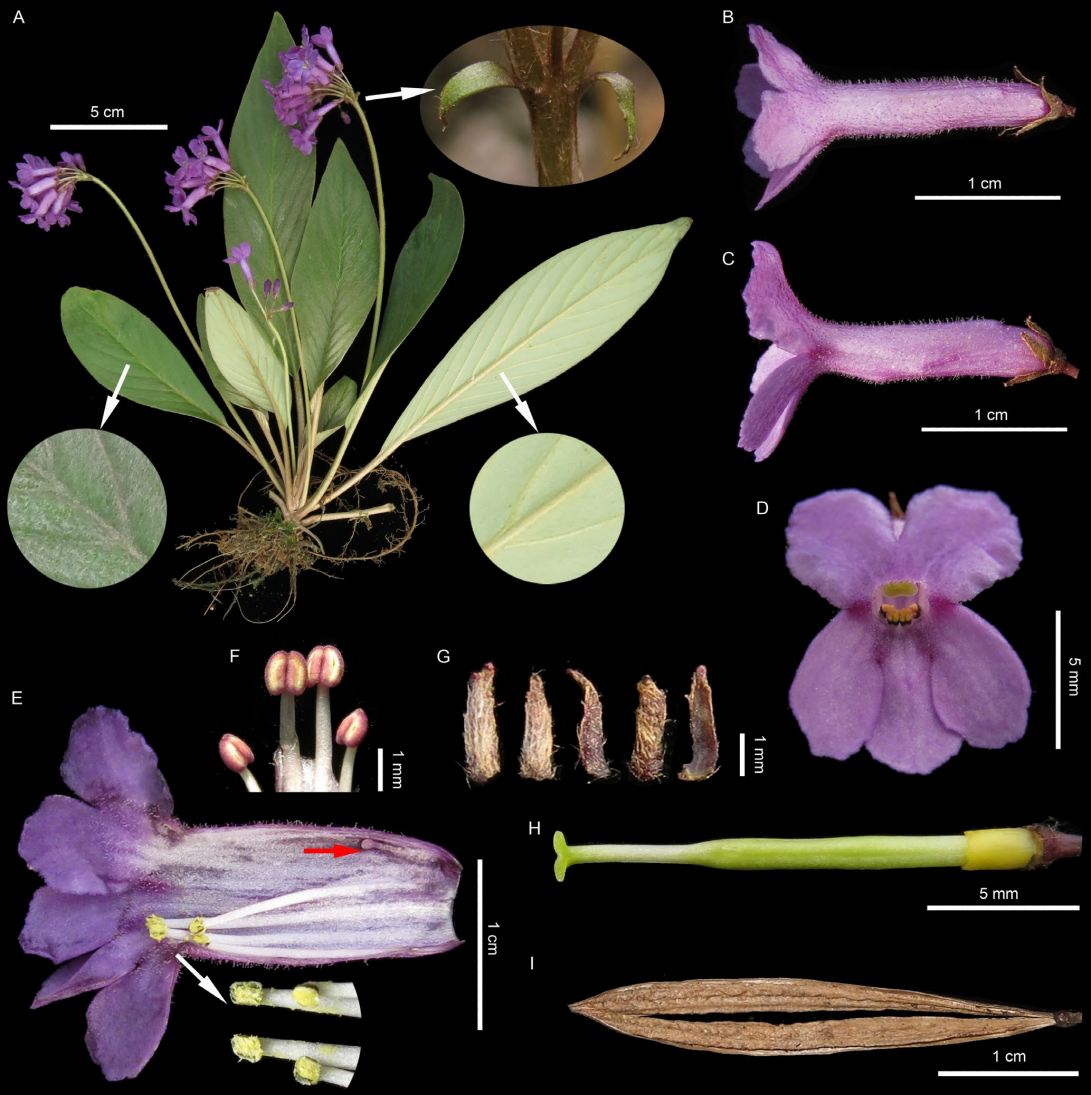
*Oreocharis nanlingensis* sp. nov. (A) Habit. (B) Flowers in top view. (C) Flowers in side view. (D) Flowers in front view. (E) Opened flower showing stamens, staminodes, and corolla inside. (F) Not yet dehiscing anthers. (G) Calyx. (H) Pistil. (I) Dehiscing fruit. All Photos were taken from its holotype locality.

**Type:** CHINA. Jiangxi: Jinggangshan, Ciping Town, Liujiaping, alt. 640 m, 114°10′9″ E, 26°33′10″ N, 16 April 2018 (flowering), L.H. Yang and H.H. Kong *YLH475*.

#### 4.1.1 Diagnosis

*Oreocharis nanlingensis* is similar to *O. crispata* , but can be distinguished from the latter by its smooth leaf blade with entire margin (vs. rugose leaf blade with crispate and irregularly dentate margin), longer corolla tube (1.6–1.7 cm vs. ca 1.3 cm), and pistil (1.5–1.6 cm vs. ca 1.1 cm), and bilobed stigma (vs. disc‐shaped).

#### 4.1.2 Description

Perennial herb. Rhizome inconspicuous. Leaves in basal rosette, 8–26; leaf blade thick chartaceous, lanceolate, sometimes slightly falcate, 6.7–25 × 1.4–5.9 cm, margin entire, apex acute, base cuneate to oblique; adaxial surface densely appressed pubescent, abaxial surface wooly along the veins; lateral veins 9–15 on each side, inconspicuous adaxially, prominent abaxially; petiole 1–7 cm long, ca. 4 mm in diameter, densely brown wooly. Cymes 4–15, per cyme 10–18 flowered; peduncle 9–19 cm long, 2 mm in diameter, sparsely wooly; bracts 2, opposite, narrowly ovate to lanceolate, 6–8 × 3–4 mm, acuminate at apex, margin entire, abaxial surface wooly, adaxial surface glabrous; pedicel 0.9–1.4 cm long, glabrescent. Calyx 5 parted to base, lobes narrowly triangular, reddish brown, 3–3.5 × 0.8–1 mm, acute at apex, margin entire, abaxial surface densely wooly, adaxial surface glabrous. Corolla purple to reddish purple, salverform, 2.1–2.4 cm long, outside glandular‐pubescent, inside glabrous; tube tubular, slightly constricted at throat, 1.6–1.7 cm long, 4 mm in diameter; limb 2‐lipped, both surface pubescent; adaxial lip 2‐lobed, lobes broadly oblong, ca. 4 mm in diameter, apex rounded, margin undulate terminally; abaxial lip 3‐lobed, lobes oblong, 5–6 × 4 mm, apex rounded, margin undulate terminally, two lateral lobes slightly overlapping with the middle one. Stamens 4, adnate at 5–6 mm above the corolla tube base; filament linear, glabrous; adaxial 2 stamens ca.7 mm long, abaxial 2 stamens 9–10 mm long; anthers dorsifixed, free, elliptic, dehiscing longitudinally, glabrous. Staminode 1, adnate at 3–4 mm above the corolla tube base, 1–2 mm long. Pistil 1.5–1.6 cm long, glabrous; ovary linear, 1–1.1 cm long; style 5–6 mm long, stigma bilobed, lobes oblong, ca. 1 × 0.8 mm. Disk ring‐like, 2 mm in height, 1 mm in diameter. Capsule linear, 3–3.5 cm long, dehiscing loculicidally; valves 2. Seeds not seen.

#### 4.1.3 Phenology

Flowering from March to April, and fruiting from April to May.

#### 4.1.4 Etymology

The specific epithet is derived from the geographical distribution of the new species. The Chinese name given is Nan Ling Ma Ling Ju Tai (南岭马铃苣苔).

#### 4.1.5 Distribution and Habitat

*Oreocharis nanlingensis* is currently found in several localities within the broader Nanling mountains region (Figure 1), spanning southwestern Jiangxi, southern Hunan, northern Guangdong, and eastern Guangxi provinces in China. Plants of this species typically grow on shady rock outcrops at elevations between ca. 200 to 1000 m.

#### 4.1.6 Paratypes

CHINA. Guangdong: Yingde, Shimentai National Nature Reserve, alt. 390 m, 113°17′50.39″ E, 24°26′16.26″ N, 20 April 2018, L.H. Yang and H.H. Kong *YLH494* (IBSC!); Ruyuan, Dabu Town, Guangdong Cayon, alt. 525 m, 113°7′30.3″ E, 24°31′1.36″ N, 15 January 2019, L.H. Yang et al. *YLH981* (IBSC!); ibid., 12 June 2025 X.Z. Shi and N.N. Peng *SXZ397* (IBSC!); Shixing, Siqian Town, Chebaling National Nature Reserve, alt. 458 m, 114°11′34.34″ E, 24°42′11.72″ N, 10 April 2020, L.H. Yang and J.Y. Wang *YLH1054* (IBSC!); Shixing, Luoba Town, Shisun Park, alt. 196 m, 114°15′26.65″ E, 24°47′28.83″ N, 17 July 2025, X.Z. Shi et al. *SXZ470* (IBSC!); Guangxi: Jinxiu, Jinxiu Town, Lianhuashan mountains, alt. 1000 m, 110°6′32.22″ E, 24°9′14.34″ N, 8 April 2016, P.W. Li *LPW2016010* (PE!); Jiangxi: Jinggangshan, Road from Jinggangshan Nature Reserve to Xiping, alt. 660 m, 114°13′45.6″ E, 26°34′54.6″ N, 25 August 2016, P.W. Li *LPW2016092* (PE!); Hunan: Yanling, Taoyuandong, alt. 859 m, 114°2′24.16″ E, 26°29′45.41″ N, 9 April 2014, W.B. Liao et al. *LXP13‐5295* (SYS!).

### 4.2 *Oreocharis argyreia* Chun ex K.Y. Pan in Pan (1987, 283)

**Type:** CHINA. Guangxi, Jingxi, Biaolin Town, 22 August 1935, X.P. Gao *55553* (IBSC!; isotypes: A [digital image!], MO [digital image!]).

= *O. argyreia var. angustifolia* K.Y. Pan in Acta Phytotax. Sin. 25: 285. 1987, syn. nov. **Type:** CHINA. Guangxi, Shangsi, Shiwandashan mountains, Denglong Village (belong to Shiwandashan mountains National Park, now), 6 September 1934, W.T. Tsang 24218 (IBSC!; isotypes: A [digital image!], NY [digital image!], MO[digital image!]).

#### 4.2.1 Amended Description

Perennial herbs. Rhizome inconspicuous. Leaves in basal rosette; petiole 3–13 cm long, with densely villous or pubescent; leaf blade elliptic, ovate or lanceolate, 5.2–15.1 × 2.3–5.5 cm, adaxially densely white villous or pubescent, abaxially white villous or pubescent along veins; base cuneate to subrounded, margin nearly entire, apex acute to acuminate; lateral veins 5–8 on each side of midrib. Cymes 1–5, axillary, 2–22‐flowered; peduncle 10–20 cm long, light brown to white pubescent or villous; bracts 2, lanceolate, 0.8–1.3 × 1.5–2 mm, pubescent or villous, margin entire. Pedicel 0.9–2.5 cm long, pubescent or villous. Calyx 5‐lobed near base, lobes equal, narrowly lanceolate to narrowly triangular, 4–8 × 1–1.5 mm long, apex acuminate, margin entire or denticulate, abaxial surface pubescent or villous. Corolla blue‐purple, 1.8–2.3 cm, outside sparsely pubescent to glabrescent; tube nearly cylindric, gradually ampliative from base to mouth, 1.4–2 cm long, 4–6 mm in diameter; limb slightly 2‐lipped; adaxial lip 2‐lobed near middle, lobes semi‐orbicular, 1.5–2 × 2–2.5 mm, apex rounded, margin entire; abaxial lip 3‐lobed near base, lobes semi‐orbicular, 3.5–5.5 × 2.5–3 mm, apex rounded, margin entire. Stamens 4, free, adaxial stamens 0.9–1.1 mm long, adnate to 5–6 mm above corolla base, abaxial stamens 1–1.2 long, adnate to 6–7 mm above corolla base; filaments slender, slightly flattened, glabrous; anthers oblong, dorsifixed, 2‐loculed, dehiscing longitudinally, glabrous; staminode 1, ca. 1.2 mm, adnate to ca. 1 mm above corolla base. Disc ca. 1.2 mm, shallowly 5 lobed to entire. Pistil 0.9–1.8 cm, glabrous; ovary linear, 0.7–1.5 cm; style 2–3 mm; stigma disc‐shaped. Capsule 3–4.5 cm, dehiscing loculicidally to base.

#### 4.2.2 Distribution and Habitat

*Oreocharis argyreia* is currently known from Guangxi and Guangdong provinces in China, where it occurs along the Shiwandashan, Dayaoshan, and Nanling mountains (Figure 1). Plants grow on moist rock surfaces in evergreen forests at elevation between ca. 200–1200 m.

#### 4.2.3 Specimens Examined

CHINA. Guangdong: Lianshan, 6 Jul 2024, L.H. Yang et B.Q. He *YLH1325* (IBSC); Ruyuan, 7 Sep 1935, J.X. Zhong 11007 (IBSC); 17 Nov 1936, Y. Li 2132 (IBSC); 29 Nov 1936, Y. Li 2132 (IBK); 27 Mar 1934, X.P. Gao 53973 (PE, IBSC, NAS, IBK, SN). Guangxi: Debao, 2 Dec 1958, Z.T. Li 602103 (IBK); 6 Sep 2016, *Debao Survey Team 451024160906007LY* (IBK); Guanyang, 13 Jun 2015, *Guanyang Survey Team 450327150613058LY* (IBK); Jinxiu, 26 Sep 1959, Q.H. Lü 4228 (IBSC, KUN, PE); 30 Oct 1972, X.F. Deng 11,491 (IBK); 12 Oct 1936, Z. Huang 40019 (IBSC); 12 Oct 1936, Z. Huang 40028 (IBSC, IBK); 15 Oct 1936, Z. Huang 40166 (IBSC); 24 Jul 1936, Z. Huang 39652 (IBSC); 11 Oct 2017, F.P. Liu et al. *lfp2017036* (PE); 8 Apr 2016, P.W. Li *LPW2016009* (PE); 22 Sep 1959, Q.H. Lü 4453 (PE, KUN); 21 Jul 1959, Q.H. Lü 4453 (IBSC); 30 May 2014, C.Y. Feng FCY2014026 (PE); 30 May 2014, C.Y. Feng *FCY2014021* (PE); 1 Aug 1958, Y.K. Li 400,822 (IBSC, IBK); 11 Jun 1928, S.Z. Xin 458 (IBSC); 18 Feb 1924, *unknown 272* (IBSC); 22 Aug 1951, B.G. Li 368 (IBSC); 8 Sep 1981, *Dayao Mountains Comprehensive Expedition Team 10,237* (IBSC, IBK); 29 Nov 2008, W.B. Xu et al. *Liuyan0127* (KUN); 19 Aug 2012, L. Lu et al. *WH‐2012‐0879* (KUN); 13 Oct 1981, *Dayao Mountains Comprehensive Expedition Team* 11327 (IBK); 31 Oct 1981, *Dayao Mountains Comprehensive Expedition Team 12838* (IBK); 29 Aug 2014, M. Guo et al. *451324140829021LY* (GXMG); 4 Sep 2024, L.H. Yang et J.X. Li *YLH1346* (IBSC); 5 Sep 2024, L.H. Yang et J.X. Li *YLH1358* (IBSC); Mengshan, 17 Dec 1943, J.X. Zhong 85046 (IBK); Ningming, 6 Jul 2011, W.B. Xu et al. *NM748* (IBK); 8 Dec 2012, Y.D. Peng et al. *451422121208109LY* (GXMG); Shanglin, 6 Aug 1973, Z.Q. Liu Group 66947 (GXMI); 26 Aug 1958, X.Y. Huang et al. 607 (GXMI); 26 Aug 1958, Y.C. Chen 00607 (IBK); 10 Sep 2011, M.T. Liu *LMT2011012* (PE); 31 Jul 1951, C.X. Cai *5023* (IBK); Shangsi, 13 Apr 2008, N.F. Li et al. 17802 (GXMG); 22 Sep 2009, *Shiwandashan Collection Team* 908 (IBK); 16 Jul 1937, X.R. Liang 69694 (IBSC); 6 Oct 2005, Y.D. Peng et B.Y. Huang 16342 (GXMG); 2 Aug 2018, L.H. Yang et al. YLH631 (IBSC); 18 May 2025, X.Z. Shi et N.N. Peng *SXZ364* (IBSC); 14 Sep 2015, X.Y. Huang et al. *451425150914043LY* (GXMG); 13 May 1966, J.J. Wang 005338 (PE, GXMI); 11 Aug 2007, R.H. Liang lrh021 (PE); 18 May 2025, X.Z. Shi et N.N. Peng SXZ365 (IBSC); 3 Jul 2025, X.Z. Shi et N.N. Peng SXZ427 (IBSC); 4 Jul 2025, X.Z. Shi et N.N. Peng SXZ430 (IBSC); Tiandeng, 14 Sep 2015, X.Y. Huang et al. 451425150914043LY (GXMG); Wuming, 22 Aug 1951, C.X. Cai 5360 (IBSC); 5 Aug 2010, L. Wu et al. D0290 (IBK); 5 Aug 2010, L. Wu et al. D0377 (IBK); 26 Aug 2007, S.Z. Zhang et M.S. Zhou W070223 (SZG); 9 Oct 2010, X.X. Huang et al. 9846 (IBSC); 20 Oct 2022, M.X. Li et al. DMB1169 (PE); 16 Jul 2022, C.L. Su et al. DMA0112 (PE); 1 Aug 2018, L.H. Yang et al. YLH626 (IBSC); 20 May 2025, X.Z. Shi et N.N. Peng SXZ366 (IBSC); Zhaoping, 14 Aug 1957, C.Z. Jiang et M.S. Xia 4196 (IBK, IBSC); 8 Apr 1958, Y.K. Li 402493 (IBK); 24 May 2025, X.Z. Shi et N.N. Peng *SXZ374* (IBSC).

### 4.3 *Oreocharis magnidens* Chun ex K.Y. Pan in Pan (1987, 276)

**Type:** CHINA. Guangxi, Xiangzhou, Guchen, Yaoshanxincun (belong to Liuxiang, Jinxiu, Guangxi, now), 24 July 1973, Z. Huang 39651 (IBK!; isotypes: IBSC!, PE!, A [digital image!]).

= *O. auricula var. denticulata* K.Y. Pan in Acta Phytotax. Sin. 25: 276. 1987, syn. nov. **Type:** CHINA. Fujian, Yong'an, Neilu, Lianhuadong, 2 July 1959, *Fujian Expedition Team 005367* (IBSC!).

#### 4.3.1 Amended Description

Perennial herbs. Rhizome inconspicuous. Leaves in basal rosette; petiole 2–9 cm long, with densely brown wooly; leaf blade obovate to elliptic, 4–15 × 3–7 cm, adaxially white villous and bullate, abaxially densely brown wooly along veins, pubescent between veins; base cuneate and slightly oblique, margin dentate to crenate, apex subacute to rounded; lateral veins 5–9 on each side of midrib. Cymes 1–4, axillary, 8–12‐flowered; peduncle 7–20 cm long, villous; bracts 2, lanceolate, 5–6 × 1.5–3.5 mm, abaxial surface villous, margin entire. Pedicel 7–25 mm long, pubescent. Calyx 5‐lobed near base, lobes equal, lanceolate, 2.5–3 mm long, apex acute, margin entire, abaxial surface pubescent. Corolla blue‐ to red‐purple, outside sparsely pubescent; tube cylindric, constricted at throat, ca.12 mm long, base ca. 3 mm in diameter, mouth ca. 1.5 mm in diameter; limb 2‐lipped; adaxial lip 2‐lobed near base, lobes narrowly oblong, 5–6 × 1.5–2 mm, apex acute, margin entire; abaxial lip 3‐lobed near base, lobes narrowly oblong, 5–9 × 1.5–2 mm, apex acute, margin entire. Stamens 4, free, adaxial stamens ca. 7 mm long, adnate to ca. 2 mm above corolla base, abaxial stamens ca. 9 mm long, adnate to ca. 3 mm above corolla base; filaments glabrous; anthers oblong, dorsifixed, 2‐loculed, dehiscing longitudinally, glabrous; staminode 1, ca. 1.2 mm, adnate to ca. 1 mm above corolla base. Disc ca. 1.5 mm, slightly undulate. Pistil ca. 8 mm, glabrous; ovary oblong, ca. 6 mm; style ca. 2 mm long; stigma disc‐shaped. Capsule 2.6–3.6 cm, glabrescent, dehiscing loculicidally to base.

#### 4.3.2 Distribution and Habitat

*Oreocharis magnidens* is currently known from Guangxi, Guangdong, Hunan, Jiangxi, and Fujian provinces in China, where it occurs along the Dayaoshan, Nanling, Luoxiaoshan, and Wuyishan mountains (Figure 1). Plants grow on moist rock surfaces in evergreen forests at an elevation between ca. 500–1600 m.

#### 4.3.3 Specimens Examined

Guangdong: Pingyuan, 7 Apr 1957, L. Deng 4117 (IBSC); Ruyuan, 15 Jan 2019, L.H. Yang et al. YLH979 (IBSC). Guangxi: Jinxiu, 12 Jul 1934, *Faculty of Biology of Sun Yat‐sen University 23514* (IBK); 12 Oct 1936, Z. Huang 40044 (IBK); 21 Feb 1924, *Guangxi Museum 286* (IBSC); 14 Oct 1981, *Dayaoshan Mountains Comprehensive Expedition Team* 11395 (IBSC); 31 Oct 1981, *Dayaoshan Mountains Comprehensive Expedition Team* 12895 (IBSC, IBK); 22 Nov 1981, *Dayaoshan Mountains Comprehensive Expedition Team 13,375* (IBSC, IBK); 12 Jul 1934, S.Z. Xin 23514 (IBSC, IBK); 15 Jan 1928, S.Z. Xin 2107 (IBSC); 14 Oct 1981, *Dayaoshan Mountains Comprehensive Expedition Team* 810,877 (GXMI); 30 May 2014, C.Y. Feng FCY2014022 (PE); 15 Oct 1997, G.Z. Li 16872 (IBK); 20 Oct 1997, G.Z. Li 16873 (IBK); 29 Nov 2008, W.B. Xu et al. *Liuyan0122* (KUN); 11 Oct 2017, F.P. Liu et al. *lfp2017035* (PE); 4 Sep 2024, L.H. Yang et J.X. Li *YLH1347* (IBSC). Hunan: Rucheng, 22 Aug 2017, J.L. Luo et A. Liu 665 (CSFI); Guidong, 18 Apr 2018, L.H. Yang et H.H. Kong *YLH481* (IBSC); 18 Dec 2017, W.Y. Zhao et al. *LXP‐13‐25357* (SYS). Jiangxi: Luxi, 6 Nov 2015, W.Z. Tan et al. *LXP‐13‐08259* (SYS); Anfu, 15 Apr 2018, L.H. Yang et H.H. Kong *YLH471* (IBSC); Shangyou, 30 May 2015, W.Y. Zhao et al. *LXP‐13‐12012* (SYS); Chongyi, 28 Aug 2017, W.Y. Zhao et al. *LXP‐13‐24416* (SYS). Fujian: Yong'an, 10 Apr 2018, L.H. Yang et H.H. Kong *YLH452* (IBSC); Xinluo, 3 Jul 2017, L.H. Yang et B. Pan *YLH394* (IBSC).

## Author Contributions

**Xi‐Zuo Shi:** data curation (lead), formal analysis (lead), investigation (equal), writing – original draft (equal), writing – review and editing (equal). **Zhi‐Xia Sun:** data curation (supporting), funding acquisition (supporting), investigation (supporting), writing – review and editing (supporting). **Jia‐Xin Fu:** data curation (supporting), investigation (supporting). **Li‐Hua Yang:** conceptualization (lead), funding acquisition (lead), investigation (lead), supervision (lead), validation (lead), writing – original draft (equal), writing – review and editing (lead).

## Conflicts of Interest

The authors declare no conflicts of interest.

## Supporting information


**Figure S1:** Types of *Oreocharis argyreia var. argyreia* . (A) Holotype of *O. argyreia var. argyreia* (X.P. Gao 55553, IBSC!). (B, C) Isotypes of *O. argyreia var. argyreia* (X.P. Gao 55553, MO [digital image!], A[digital image!]). (D) One of the paratypes of *O. argyreia var. argyreia* (Z. Huang 40019, IBSC!).

**Figure S2:** Types of *Oreocharis argyreia var. angustifolia* . (A) Holotype (W.T. Tsang 24218, IBSC!). (B) Isotype (A [digital image!]). (C) One of the paratypes (Y.K. Li 402493, IBK!). (D) One of the paratypes (J.J. Wang 5338, PE!).

**Figure S3:** Types of *Oreocharis auricula* var. *denticulate* and *O. magnidens* . (A) Holotype of *O. auricula* var. *denticulate* (*Fujian Expedition Team 005367*, IBSC!). (B) Holotype of *O. magnidens* (C. Huang 39651, IBK!). (C, D) Isotypes of *O. magnidens* (Z. Huang 39651, A [digital image!], IBSC!).

**Figure S4:** Types of *Oreocharis nanlingensis* sp. nov. (A) Holotype (China: Jiangxi, Jinggangshan, L.H. Yang and H.H. Kong *YLH475*, IBSC!). (B) One of the paratypes (China: Hunan, Yanling, W.B. Liao et al. *LXP13‐5295*, SYS!). (C) One of the paratypes (China: Guangxi, Jinxiu, P.W. Li *LPW2016010*, PE!). (D) One of the paratypes (China: Guangdong, Yingde, LH. Yang et H.H. Kong *YLH494*, IBSC!).

**Table S1:** Morphological measurements.
**Table S2:** GenBank accession numbers and voucher information for the eight nuclear loci used in this study.
**Table S3:** Results of Tukey's HSD post hoc test for pairwise comparisons of leaf measurements among taxa.

## Data Availability

The DNA sequences generated in the present study have been deposited in the National Center for Biotechnology Information (NCBI) database. The accession numbers and the information on the voucher specimens are available in Table S2. The voucher specimens of the new species were housed in IBSC.
