# Supplementary figures and images for: Integrating Morphological and Molecular Evidence Reveals a New Species and Two Synonyms in Oreocharis (Gesneriaceae)
Source: Ecol Evol. 2026 Apr 6;16(4):e73380. doi: 10.1002/ece3.73380 (PMC13053118; doi:10.1002/ece3.73380)

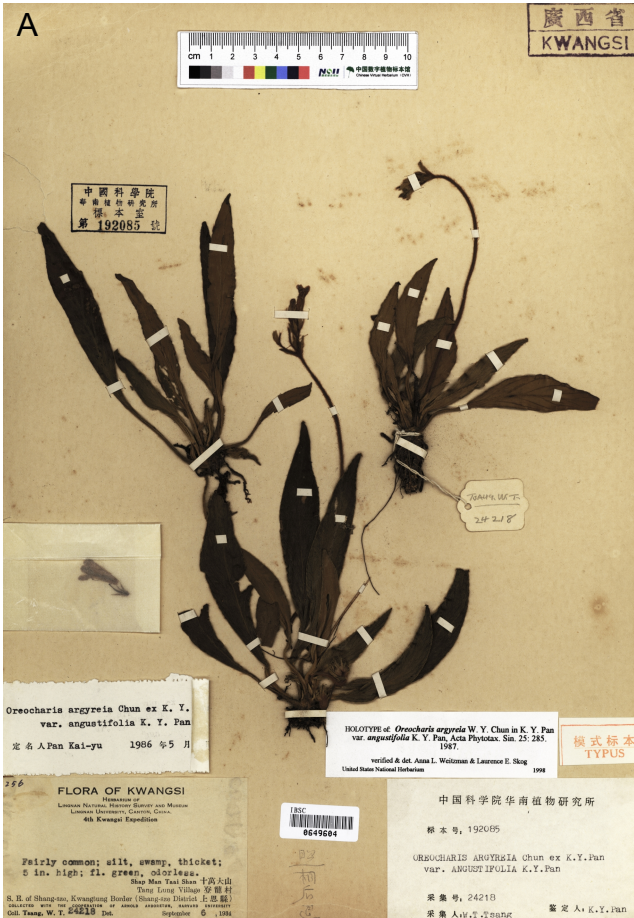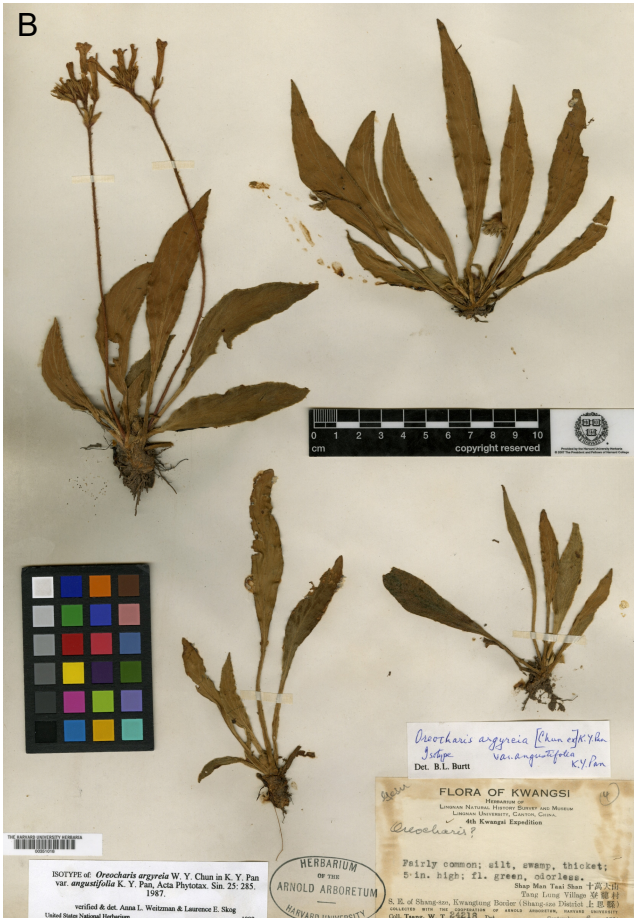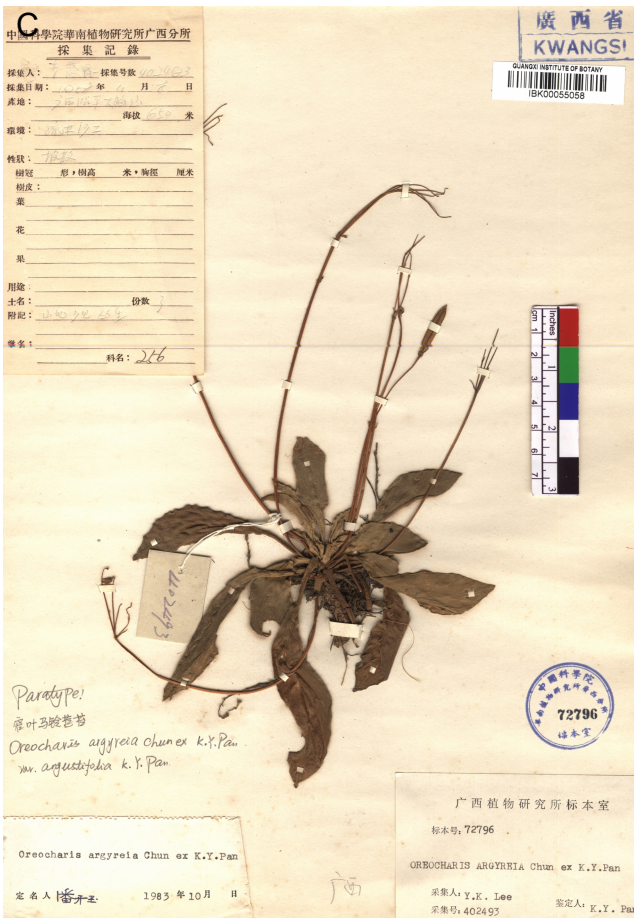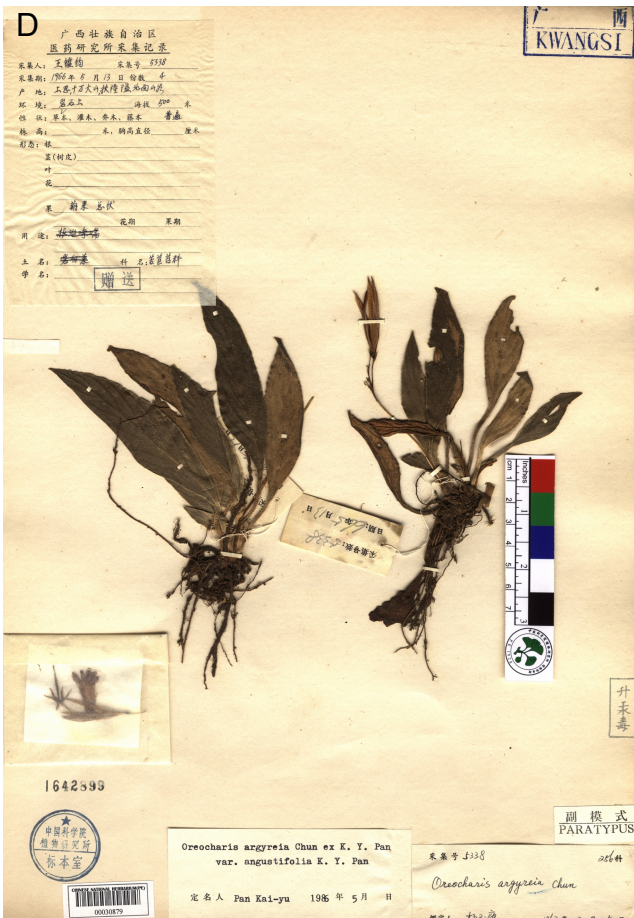

Supplement: Supplementary file 2 — Figure S2: Types of Oreocharis argyreia var. angustifolia . (A) Holotype (W.T. Tsang 24218, IBSC!). (B) Isotype (A [digital image!]). (C) One of the paratypes (Y.K. Li 402493, IBK!). (D) One of the paratypes (J.J. Wang 5338, PE!). [file ECE3-16-e73380-s003.pdf]
