# Supplementary material for: Integrating Morphological and Molecular Evidence Reveals a New Species and Two Synonyms in Oreocharis (Gesneriaceae)
Source: Ecol Evol. 2026 Apr 6;16(4):e73380. doi: 10.1002/ece3.73380 (PMC13053118; doi:10.1002/ece3.73380)
Supplement: Supplementary file 1 — Figure S1: Types of Oreocharis argyreia var. argyreia . (A) Holotype of O. argyreia var. argyreia (X.P. Gao 55553, IBSC!). (B, C) Isotypes of O. argyreia var. argyreia (X.P. Gao 55553, MO [digital image!], A[digital image!]). (D) One of the paratypes of O. argyreia var. argyreia (Z. Huang 40019, IBSC!). [file ECE3-16-e73380-s004.pdf]

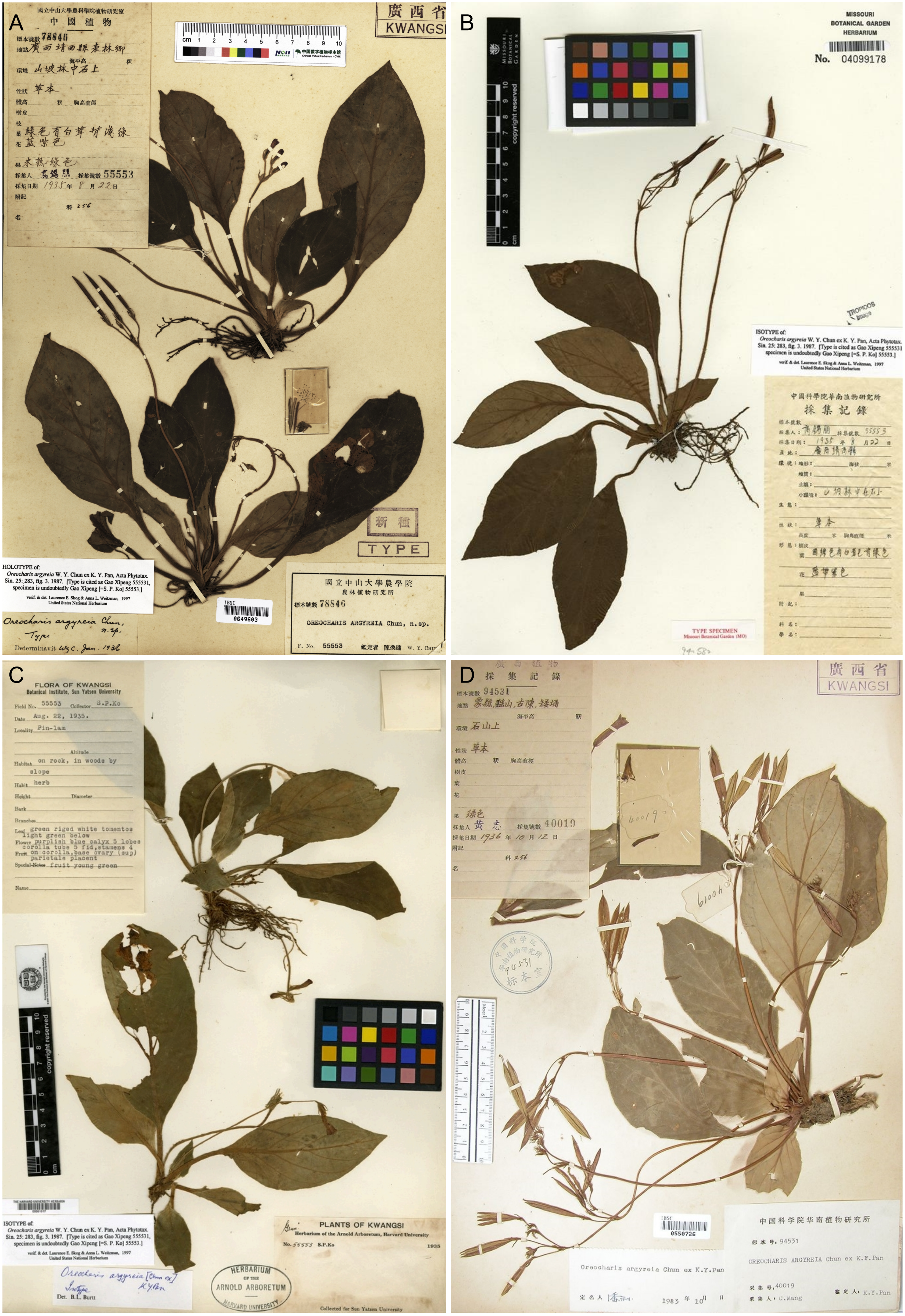
A
廣西省
KWANGSI
國立中山大學農科學院植物研究室
中國植物
標本號數 78846
地點 廣西靖西縣表林鄉
環境 山坡林中石上
性狀 草本
葉 綠色有白茸背淺綠
花 藍紫色
果 未熟綠色
採集人 高錫朋 採集號數 55553
採集日期 1935 年 8 月 22 日
科 256
新種
TYPE
HOLOTYPE of:
Oreocharis argyreia W. Y. Chun ex K. Y. Pan, Acta Phytotax. Sin. 25: 283, fig. 3. 1987. [Type is cited as Gao Xipeng 555531, specimen is undoubtedly Gao Xipeng [=S. P. Ko] 55553.]
verif. & det. Laurence E. Skog & Anna L. Weitzman, 1997
United States National Herbarium
IBSC
0649603
國立中山大學農學院
農林植物研究所
標本號數 78846
OREOCHARIS ARGYREIA Chun, n.sp.
F. No. 55553 鑑定者 陳煥鏞 W. Y. Chun
Oreocharis argyreia Chun, n. sp.
Type
Determinavit W.Y.C. Jan. 1936
B
MISSOURI
BOTANICAL GARDEN
HERBARIUM
No. 04099178
copyright reserved
TROPICOS image
ISOTYPE of:
Oreocharis argyreia W. Y. Chun ex K. Y. Pan, Acta Phytotax. Sin. 25: 283, fig. 3. 1987. [Type is cited as Gao Xipeng 555531, specimen is undoubtedly Gao Xipeng [=S. P. Ko] 55553.]
verif. & det. Laurence E. Skog & Anna L. Weitzman, 1997
United States National Herbarium
中國科學院華南植物研究所
採集記錄
草本
TYPE SPECIMEN
Missouri Botanical Garden (MO)
C
FLORA OF KWANGSI
Botanical Institute, Sun Yatsen University
Field No. 55553 Collector S.P.Ko
Date Aug. 22, 1935.
Locality Pin-lam
Habitat on rock, in woods by slope
Habit herb
Leaf green riged white tomentose light green below
Flower purplish blue calyx 5 lobes corolla tube 5 fid, stamens 4 on corolla, base ovary (sup) parietale placent
Special Notes fruit young green
copyright reserved
ISOTYPE of:
Oreocharis argyreia W. Y. Chun ex K. Y. Pan, Acta Phytotax. Sin. 25: 283, fig. 3. 1987. [Type is cited as Gao Xipeng 555531, specimen is undoubtedly Gao Xipeng [=S. P. Ko] 55553.]
verif. & det. Laurence E. Skog & Anna L. Weitzman, 1997
United States National Herbarium
PLANTS OF KWANGSI
Herbarium of the Arnold Arboretum, Harvard University
No. 55553 S.P.Ko 1935
Collected for Sun Yatsen University
HERBARIUM OF THE ARNOLD ARBORETUM HARVARD UNIVERSITY
Oreocharis argyreia [Chun ex] K.Y.Pan
Isotype
Det. B.L. Burtt
D
廣西省
KWANGSI
採集記錄
標本號數 94531
環境 石山上
性狀 草本
果 綠色
採集人 黃志 採集號數 40019
採集日期 1936 年 10 月 12 日
科 256
40019
IBSC
0550726
中国科学院华南植物研究所
标本号 94531
OREOCHARIS ARGYREIA Chun ex K.Y.Pan
采集号 40019
采集人 C.Wang
鉴定人 K.Y.Pan
Oreocharis argyreia Chun ex K.Y.Pan
定名人
1983 年 10 月
