# Supplementary material for: Integrating Morphological and Molecular Evidence Reveals a New Species and Two Synonyms in Oreocharis (Gesneriaceae)
Source: Ecol Evol. 2026 Apr 6;16(4):e73380. doi: 10.1002/ece3.73380 (PMC13053118; doi:10.1002/ece3.73380)
Supplement: Supplementary file 3 — Figure S3: Types of Oreocharis auricula var. denticulate and O. magnidens . (A) Holotype of O. auricula var. denticulate (Fujian Expedition Team 005367, IBSC!). (B) Holotype of O. magnidens (C. Huang 39651, IBK!). (C, D) Isotypes of O. magnidens (Z. Huang 39651, A [digital image!], IBSC!). [file ECE3-16-e73380-s001.pdf]

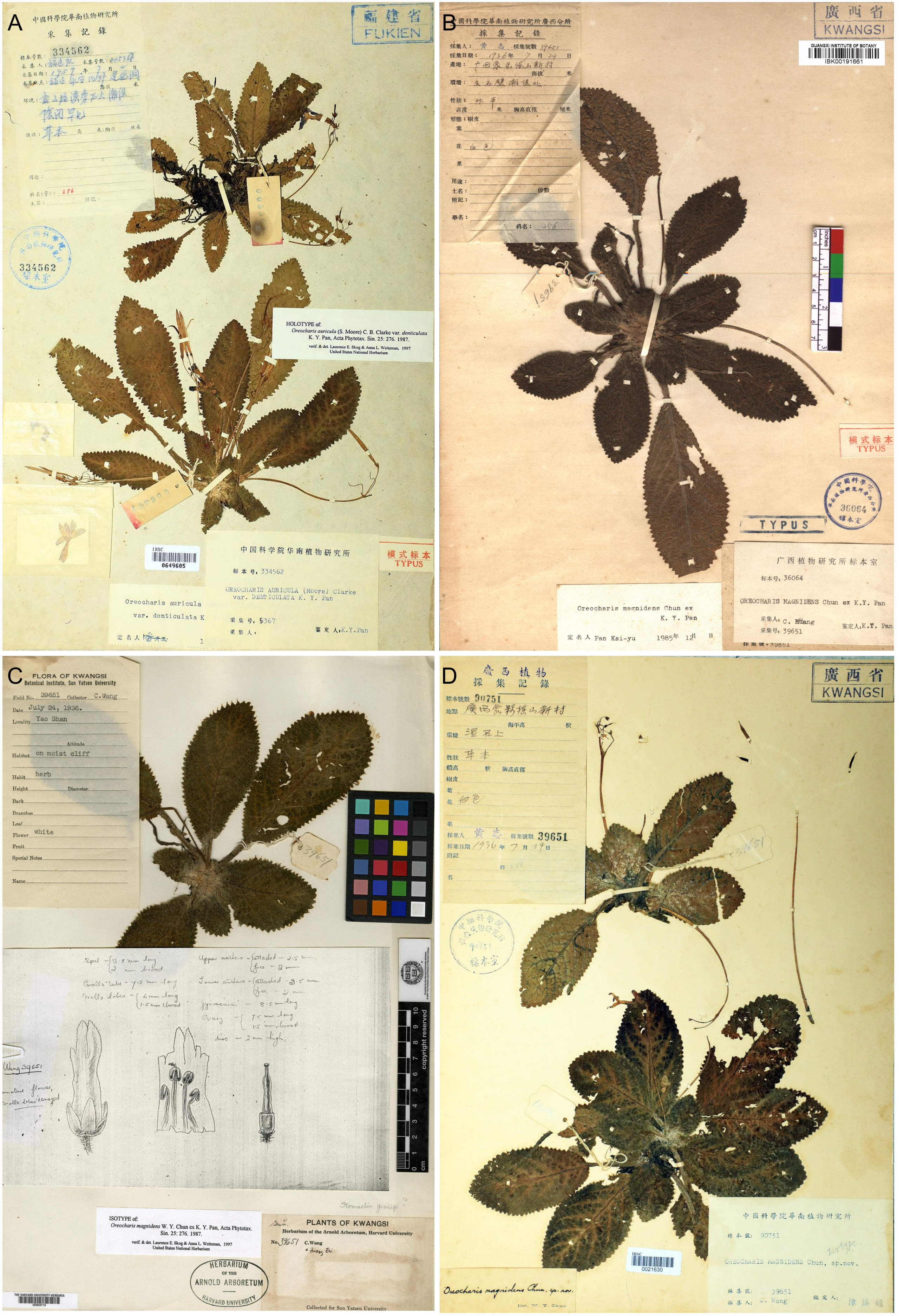
A
福建省
FUKIEN
334562
HOLOTYPE of:
Oreocharis auricula (S. Moore) C. B. Clarke var. denticulata K. Y. Pan, Acta Phytotax. Sin. 25: 276. 1987.
verif. & det. Laurence E. Skog & Anna L. Weitzman, 1997
United States National Herbarium
中国科学院华南植物研究所
标本号: 334562
OREOCHARIS AURICULA (Moore) Clarke var. DENTICULATA K. Y. Pan
采集号: 5367
鉴定人: K.Y.Pan
模式标本
TYPUS
Oreocharis auricula var. denticulata K
IBSC
0649605
B
中國科學院華南植物研究所廣西分所
採 集 記 錄
廣西省
KWANGSI
GUANGXI INSTITUTE OF BOTANY
IBK00191661
模式标本
TYPUS
TYPUS
36064
广西植物研究所标本室
标本号: 36064
OREOCHARIS MAGNIDENS Chun ex K.Y. Pan
采集人: C. Huang
采集号: 39651
鉴定人: K.Y. Pan
Oreocharis magnidens Chun ex K. Y. Pan
定名人 Pan Kai-yu 1985年 12月
C
FLORA OF KWANGSI
Botanical Institute, Sun Yatsen University
Field No. 39651 Collector C. Wang
Date July 24, 1936.
Locality Yao Shan
Habitat on moist cliff
Habit herb
Flower white
copyright reserved
ISOTYPE of:
Oreocharis magnidens W. Y. Chun ex K. Y. Pan, Acta Phytotax. Sin. 25: 276. 1987.
verif. & det. Laurence E. Skog & Anna L. Weitzman, 1997
United States National Herbarium
PLANTS OF KWANGSI
Herbarium of the Arnold Arboretum, Harvard University
No. 39651 C.Wang
HERBARIUM OF THE ARNOLD ARBORETUM HARVARD UNIVERSITY
Collected for Sun Yatsen University
D
廣西植物
採 集 記 錄
廣西省
KWANGSI
標本號數 90751
採集號數 39651
中國科學院華南植物研究所
標本號: 90751
OREOCHARIS MAGNIDENS Chun, sp.nov.
採集號: 39651
採集人: C. Wang
IBSC
0021630
Oreocharis magnidens Chun, sp. nov.
Det. W. Y. Chun
