# Supplementary material for: Integrating Morphological and Molecular Evidence Reveals a New Species and Two Synonyms in Oreocharis (Gesneriaceae)
Source: Ecol Evol. 2026 Apr 6;16(4):e73380. doi: 10.1002/ece3.73380 (PMC13053118; doi:10.1002/ece3.73380)
Supplement: Supplementary file 4 — Figure S4: Types of Oreocharis nanlingensis sp. nov. (A) Holotype (China: Jiangxi, Jinggangshan, L.H. Yang and H.H. Kong YLH475, IBSC!). (B) One of the paratypes (China: Hunan, Yanling, W.B. Liao et al. LXP13‐5295, SYS!). (C) One of the paratypes (China: Guangxi, Jinxiu, P.W. Li LPW2016010, PE!). (D) One of the paratypes (China: Guangdong, Yingde, LH. Yang et H.H. Kong YLH494, IBSC!). [file ECE3-16-e73380-s002.pdf]

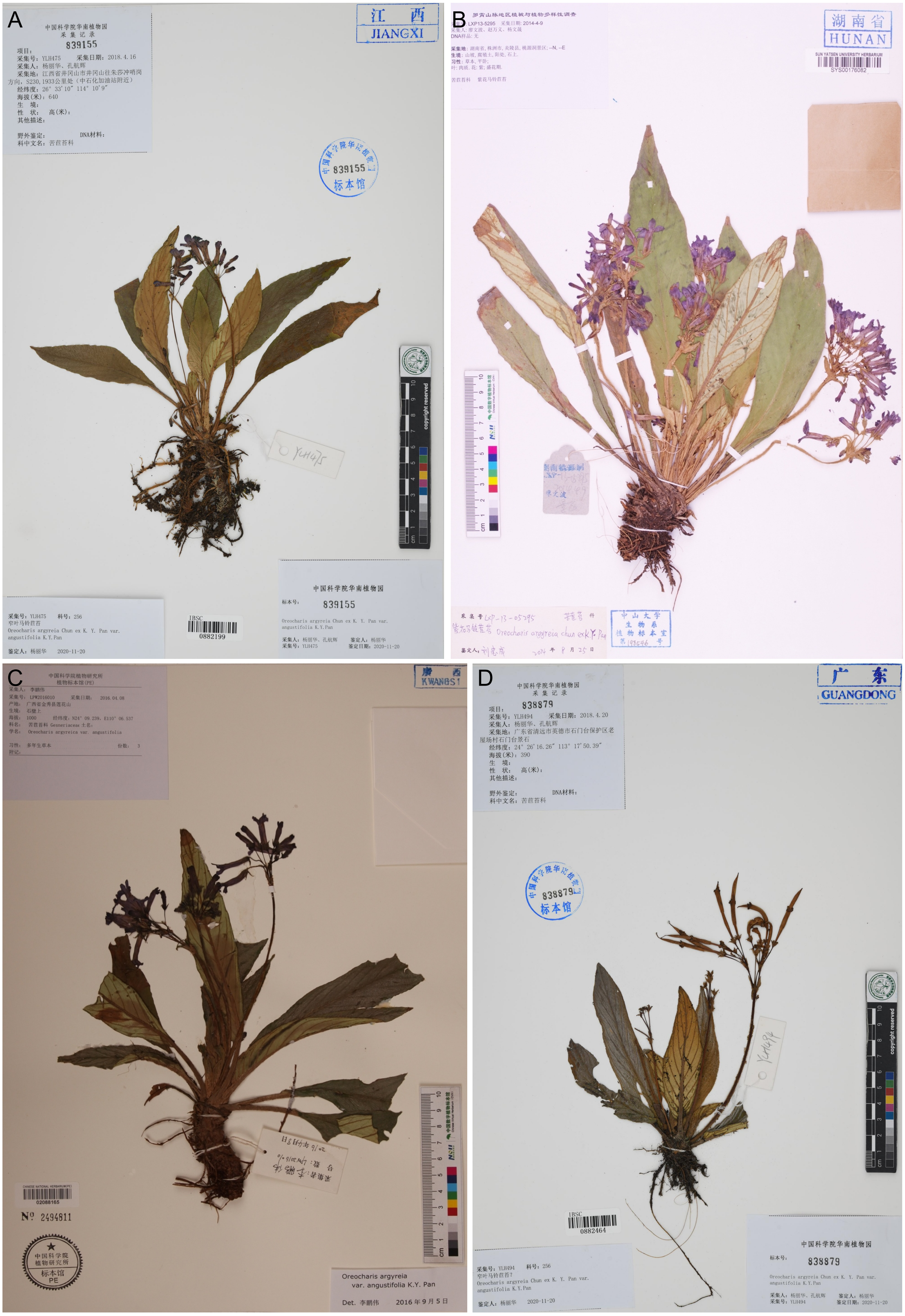
A
中国科学院华南植物园
采 集 记 录
839155
项目:
采集号: YLH475 采集日期: 2018. 4. 16
采集人: 杨丽华、孔航辉
采集地: 江西省井冈山市井冈山往朱莎冲哨岗方向, S230, 1933公里处 (中石化加油站附近)
经纬度: 26° 33′ 10″ 114° 10′ 9″
海拔(米): 640
生 境:
性 状: 高(米):
其他描述:
野外鉴定: DNA材料:
科中文名: 苦苣苔科
江 西
JIANGXI
中国科学院华南植物园标本馆 839155
copyright reserved
采集号: YLH475 科号: 256
窄叶马铃苣苔
Oreocharis argyreia Chun ex K. Y. Pan var. angustifolia K. Y. Pan
鉴定人: 杨丽华 2020-11-20
IBSC 0882199
中国科学院华南植物园
标本号: 839155
Oreocharis argyreia Chun ex K. Y. Pan var. angustifolia K. Y. Pan
采集人: 杨丽华、孔航辉 鉴定人: 杨丽华
采集号: YLH475 鉴定日期: 2020-11-20
B
罗霄山脉地区植被与植物多样性调查
LXP13-5295 采集日期: 2014-4-9
湖南省
HUNAN
SUN YATSEN UNIVERSITY HERBARIUM
SYS00176082
苦苣苔科 紫花马铃苣苔
Oreocharis argyreia Chun ex K. Y. Pan
中山大学生物系植物标本室
C
中国科学院植物研究所
植物标本馆 (PE)
采集人: 李鹏伟
采集号: LPW2016010 采集日期: 2016. 04. 08
产地: 广西省金秀县莲花山
生境: 石壁上
海拔: 1000 经纬度: N24° 09. 239, E110° 06. 537
科名: 苦苣苔科 Gesneriaceae 土名:
学名: Oreocharis argyreica var. angustifolia
习性: 多年生草本 份数: 3
附记:
广 西
KWANGSI
CHINESE NATIONAL HERBARIUM (PE)
02088165
No 2494811
中国科学院植物研究所标本馆 PE
Oreocharis argyreia var. angustifolia K.Y. Pan
Det. 李鹏伟 2016 年 9 月 5 日
D
中国科学院华南植物园
采 集 记 录
838879
项目:
采集号: YLH494 采集日期: 2018. 4. 20
采集人: 杨丽华、孔航辉
采集地: 广东省清远市英德市石门台保护区老屋场村石门台景石
经纬度: 24° 26′ 16. 26″ 113° 17′ 50. 39″
海拔(米): 390
生 境:
性 状: 高(米):
其他描述:
野外鉴定: DNA材料:
科中文名: 苦苣苔科
广 东
GUANGDONG
中国科学院华南植物园标本馆 838879
copyright reserved
IBSC 0882464
采集号: YLH494 科号: 256
窄叶马铃苣苔?
Oreocharis argyreia Chun ex K. Y. Pan var. angustifolia K. Y. Pan
鉴定人: 杨丽华 2020-11-20
中国科学院华南植物园
标本号: 838879
Oreocharis argyreia Chun ex K. Y. Pan var. angustifolia K. Y. Pan
采集人: 杨丽华、孔航辉 鉴定人: 杨丽华
采集号: YLH494 鉴定日期: 2020-11-20
